# Etoposide‐based treatment of adult HLH is associated with high biochemical response but poor survival outcomes

**DOI:** 10.1002/jha2.57

**Published:** 2020-07-14

**Authors:** Matthew C. Nicholson, Leonie Naeije, Anna R. Hayden, Andre Mattman, David Dix, Luke Y.C. Chen

**Affiliations:** ^1^ Division of Hematology, Department of Medicine University of British Columbia Vancouver BC Canada; ^2^ Division of Hematology, Oncology and Bone Marrow Transplant, Department of Paediatrics University of British Columbia Vancouver BC Canada; ^3^ Department of Pathology and Laboratory Medicine University of British Columbia Vancouver Canada; ^4^ Centre for Health Education Scholarship University of British Columbia Vancouver BC Canada

**Keywords:** etoposide, ferritin, hemophagocytic, HLH, soluble interleukin‐2 receptor

## Abstract

Etoposide‐based treatment is the standard of care for adult HLH in many centers, yet there remains a paucity of data regarding treatment outcomes. We conducted a retrospective study of 23 adults treated with etoposide‐based therapy compared to 10 pediatric HLH cases at a single center. At diagnosis, the median serum ferritin was 20,071 µg/L and 937 µg/L in adults and children, respectively; median sIL‐2r was 14,524 U/mL and 4,478 U/mL. Biochemical response to treatment was high, with 21/23 adults achieving >75% reduction in serum ferritin, but one year survival was only 7/21 compared to 7/10 in pediatric cases.

Treatment and outcomes in adult hemophagocytic lymphohistiocytosis (HLH) is poorly understood, with reported mortality ranging from 20 to nearly 90% [1]. This variability in outcomes is likely related to heterogeneity of disease, with macrophage activation syndrome due to autoimmune disease and non‐Epstein‐Barr virus (EBV) infection triggered HLH conferring much better prognosis than HLH associated with EBV and/or malignancy [2]. The lack of a standard first‐line treatment is also a barrier to understanding outcomes. Treatment in some historical studies was based on corticosteroids, with cytotoxic agents such as etoposide and cyclophosphamide, doxorubicin, vincristine and prednisone (CHOP)‐like regimens reserved for refractory disease or lymphoma‐associated HLH [3, 4]. In the absence of prospective clinical trial data, expert consensus guidelines recommend treatment directed at associated conditions such as autoimmune disease, lymphoma, and infectious triggers, and consideration of etoposide‐based therapy based on the pediatric HLH‐94 and HLH‐2004 protocols [5, 6]. Emerging therapies such as emapalumab, an interferon‐gamma inhibitor [7, 8], and ruxolitinib, a JAK inhibitor [9], appear to have biological activity and show promise for improving outcomes. Real‐world data on outcomes of adult HLH with etoposide‐based therapy are needed as an historical comparator for future studies. To this end, we present our experience at Vancouver General Hospital (VGH) using etoposide‐based therapy, along with the pediatric experience at British Columbia Children's Hospital (BCCH) from 2011 to 2018 to further augment the published experience in adult HLH.

Forty‐eight adults were diagnosed with HLH at VGH. Twenty‐three were treated with etoposide‐based therapy according to the HLH‐94 protocol. The other 25 patients received corticosteroid monotherapy, therapy for the patients underlying trigger, or a palliative approach due to age, comorbidities, poor performance status, or a combination thereof. Over the same period, 10 pediatric patients with HLH were diagnosed and treated at BCCH. Five of the pediatric patients were treated with etoposide‐based therapy and other five received corticosteroids, cyclosporine, rituximab, or some combination thereof. Allogeneic stem cell transplant was undertaken in 3/10 pediatric patients, including one patient who received etoposide‐based therapy. Baseline characteristics and outcomes of the 23 adult patients treated with etoposide‐based therapy and the 10 pediatric patients are summarized in Table 1.

**TABLE 1:** Clinical and laboratory characteristics of adult and pediatric HLH patients

	Adults treated with etoposide‐based therapy	Paediatric patients
Number	23	10
Age (median, range)	54 (21‐80)	10 (2 months‐17 years)
Genetic testing (number of patients tested; number positive)	Three patients tested (Cincinnati panel) with no identifiable mutations	Three patients tested One patient homozygous for MUNC13‐4 gene mutation (c.753+3G > A)
Ferritin (µg/L); median, range	20 071 (3773‐321000)	937 (80‐45700)
sIL‐2r (U/mL); median, range	14 524 (2650‐39439)	4 479 (3379‐6039)
LDH (U/L); median, range	1 343 (264‐19200)	3 175 (127‐17711)
Infectious trigger? (including EBV)	11/23(48%)	4/10
EBV Positive	7/23	2/10
Etoposide‐based treatment	23/23 (From 45 total adult patients over the same time period)	5/10 HLH 2004 Protocol
Survival at 30 days	18/23 (78%)	10/10
Survival at 90 days	12/23 (52%)	10/10
Survival at one year	7/21 (29%)	7/10
Hematopoietic stem cell transplant	2/23	3/10
Median follow up (years)	3.7 (1‐4)	4.8 (1‐10)

At diagnosis all adult patients had ferritin > 3 000 µg/L and 10 patients had ferritin > 50 000 µg/L. Eleven adult patients had infection‐related HLH (48%), 10 had malignancy‐associated HLH (43%), and two had SLE‐related HLH with no additional trigger identified (9%). Seven days after etoposide treatment, 91% (21/23) of patients had a > 75% reduction in serum ferritin. The trend in ferritin response for both survivors and nonsurvivors is illustrated in Figure 1. Additionally, sIL‐2r, EBV viral loads, and LDH declined in 91% (21/23) of patients over the course of the first 7 days. The survival rate was 78% (18/23) at 30 days, 52% (12/23) at 3 months, and 29% (7/21) at 1 year. Mortality remains high due to underlying malignancies, infections, organ damage from HLH, prolonged cytopenias, and HLH relapse after treatment. One patient in our series died of sepsis acquired during a period of prolonged neutropenia following etoposide treatment. In the pediatric group, 7/10 patients survived with median follow‐up of 5 years. Two pediatric patients experienced relapsed HLH and pulmonary infection with resultant mortality less than 1 year postallogeneic stem‐cell transplant. The third death was due to pulmonary infection in a patient who did not undergo allogeneic stem cell transplant (SCT) occurring 140 days after HLH treatment. Previous studies in pediatric populations have explored how initial ferritin value and rate of decline in ferritin can predict prognosis [10].

**FIGURE 1:**
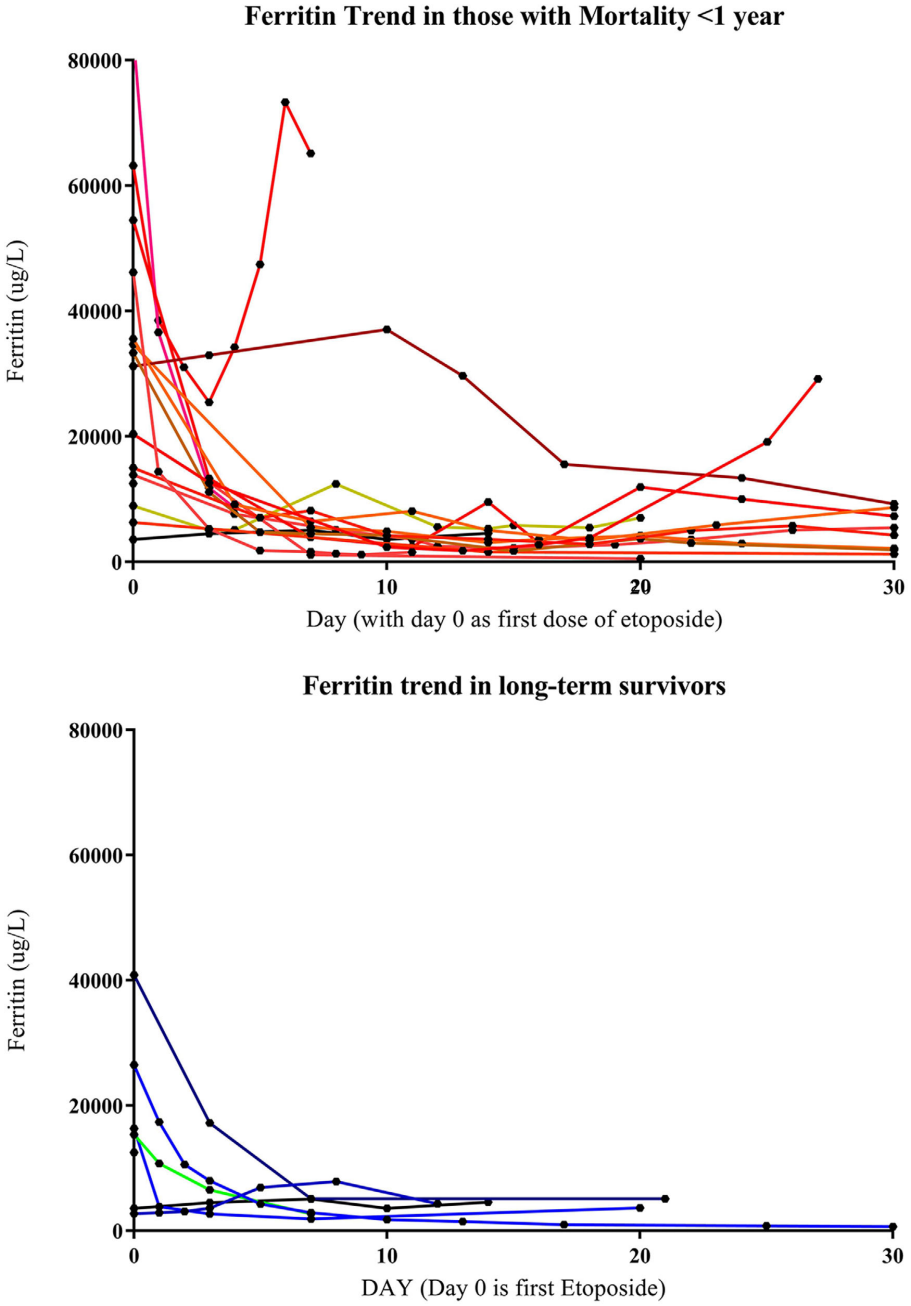
Early and sustained reduction in ferritin is seen in patients with good long‐term outcomes after etoposide therapy. Ferritin may fail to decrease or rise between treatments in those with mortality in the first year. Day zero is the first date of etoposide treatment

Patients with adult HLH present with markedly elevated ferritin and sIL‐2r, typically > 3000 µg/L and > 3000 U/mL, respectively [11, 12]. Dramatic reductions in serum ferritin, sIL‐2r, and LDH occur in nearly all patients within 1 week of treatment in this study. Patients with good long‐term outcomes from etoposide treatment are often those with a sustained low ferritin. Durable clinical remissions are much less frequent than biochemical response, underscoring the need for more effective and well‐tolerated therapies in adult HLH.

The poor survival in adult HLH of 29% at 1 year in this study aligns with other single center studies in examining etoposide‐based therapy, such a recent Mayo Clinic report wherein 17 of 31 patients achieved a partial or complete response with HLH 2004 therapy, and 1 year overall survival (OS) was 35% [13]. A large Korean study of 126 patients with secondary HLH demonstrated that those with a stable early response defined as resolution of HLH‐related symptoms and normalization of HLH‐related laboratory abnormalities, including ferritin, within 4 weeks, had much better 5 year overall survival of 87% compared to unstable early responders (5.9%) and nonresponders (0.0%, *P* < .001) [2].

Our experience in large tertiary setting over a long period of time was able to capture only a small number of patients and underscores the rarity of this disease. The small number of patients in this study prohibits definitive conclusions, and our experience should be considered hypothesis generating. Larger prospective studies examining ferritin, sIL‐2r, and other markers of immune activation such as the interferon gamma/CXCL9/10 axis over the course of treatment are needed to better understand the biology and natural history of adult HLH.

## CONFLICT OF INTEREST

No conflicts of interest are identified.
